# Genetic variation and historical breeding patterns in common bean (*Phaseolus vulgaris* L.) affect fermentation patterns by the human gut microbiome

**DOI:** 10.1038/s42003-025-09089-2

**Published:** 2025-11-26

**Authors:** Mallory J. Suhr Van Haute, Qinnan Yang, Nate Korth, Mary M. Happ, Car Reen Kok, Chenyong Miao, Jennifer L. Clarke, Kelsey Karnik, Kent M. Eskridge, Carlos A. Urrea, David L. Hyten, James C. Schnable, Devin J. Rose, Andrew K. Benson

**Affiliations:** 1 Department of Food Science and Technology, University of Nebraska, Lincoln, NE USA; 2 Nebraska Food for Health Center, University of Nebraska, Lincoln, NE USA; 3 Complex Biosystems Graduate Program, University of Nebraska, Lincoln, NE USA; 4 Department of Agronomy and Horticulture, University of Nebraska, Lincoln, NE USA; 5 Department of Statistics, University of Nebraska, Lincoln, NE USA; 6 Present Address: Department of Microbiology & Immunology, University of Michigan, Ann Arbor, MI USA; 7 Present Address: Biosciences & Biotechnology Division, Lawrence Livermore National Laboratory, Livermore, CA USA; 8 Present Address: Department of Biostatistics, University of Kentucky, Lexington, KY USA

**Keywords:** Plant genetics, Applied microbiology

## Abstract

Common beans, which contain diverse bioactive molecules, have not been systematically studied for their variation in how they affect the human gut microbiome. We measured taxonomic shifts and metabolite production of three human gut microbiomes cultured with 299 common bean cultivars under conditions that mimic the nutrient availability of the human colon. Common bean population structure (landrace and market class) had significant effects on microbiota diversity, composition, and metabolite production. Genome-wide association analysis identified seven multiple effect loci (MEL) where genetic variation in the common bean genome affected the microbiome. One MEL on chromosome *Pv*05 had impacts on the abundance of several *Lachnospiraceae* and *Ruminococcaceae*. Molecular complementation experiments suggested that variation in the biosynthesis of saponins at this MEL was the mechanism driving the variability in microbiota composition and function. This study provides innovative understanding of how genetics of common beans affects the human gut microbiome and potentially human health.

## Abstract (web summary)

Population genomic analyses identify multiple effect loci in the common bean genome that influence seed components and metabolites that consequently affect the human gut microbiome.

## Introduction

Interspecific Indirect Genetic Effects (IIGEs) are a concept in evolutionary biology and ecology that describe how the genetic traits of one species can influence the phenotype or fitness of individuals in another species, typically through ecological interactions^1^. Examples include plant-herbivore interactions^2^, predator-prey interactions^3^, and even host-microbiome interactions^4^. Here, we examine a new example of IIGEs between genetic variation and historic genetic selection in a food crop (common bean) and fermentation patterns by the human gut microbiome.

This study was motivated by the economic burden of diet-associated preventable human disease and the potential to transform our food systems to promote and enable healthier diets in a sustainable and equitable manner^5^. One approach is shifting food crop production toward species whose consumption is associated with better health outcomes. However, changing infrastructure to process these new species and the difficulty in convincing the public to change staple foods in their diets is fundamentally challenging. Another approach could be to capitalize on the substantial genetic and phenotypic variation in nutritional and health relevant traits that exist within currently cultivated crops. This approach requires identification and selection for crop genotypes that can improve human health and reduce the incidence of preventable, diet-linked human diseases.

Examples of successful efforts to leverage within-crop genetic diversity to address human health needs can be seen in biofortification, which combines crop breeding and agronomic strategies to enhance micronutrient content. Biofortification has successfully increased the content of vitamins and minerals in various food crops, which are major sources of calories in different parts of the world, resulting in improvements in diseases associated with micronutrient malnutrition^6^.

Over the past 20 years, microbiome research has shown that, beyond adequate caloric and micronutrient intake, achieving optimal health also requires food that properly feeds and supports the dense population of microbes inhabiting the human gastrointestinal tract. Indeed, the gut microbiome is now well-recognized for important contributions to human health (anatomical development, immune training, pathogen exclusion, metabolic functions)^7,8^. Research has also demonstrated the importance of dietary components in driving favorable (e.g., high-fiber diets) or unfavorable (high-fat diet) taxonomic and functional configurations of the gut microbiome, the latter of which are associated with a wide range of complex diseases including obesity, diabetes, metabolic disease, IBD, and colon cancers^9–12^.

The critical role dietary components play in controlling the structure and function of the human gut microbiome and preventing disease^13,14^ creates an opportunity to capitalize on IIGEs that likely exist between food crops and the composition and function of the human gut microbiome. Here, crop breeding and genetics could be employed to produce desirable effects on the human gut microbiome.

Common bean (*Phaseolus vulgaris* L.) is an excellent model system for studying the effects of plant-human gut microbiome IIGEs both because of the diverse array of bioactive compounds produced by beans and the widespread direct human consumption of minimally processed beans around the world. The consumption of many of the compounds in beans, including polyphenols (cyanidin-3-glucoside, kaempeferol-3-glucoside, querticin-3-glucoside, genistein), flavonoids, anthocyanins, condensed tannins, saponins, lectins, and oligosaccharides, have been associated with benefits to human health^15–18^. Several of these bioactive compounds have been studied in conjunction with human cell culture-based models^19–23^, animal model systems^24–26^, prospective human clinical studies feeding specific substrates^27–29^ and retrospective epidemiological/dietary studies^30–33^. In these studies, bioactive compounds have been shown to have significant impacts on disease incidence and predisposition, generating tremendous interest in using common beans as a means for dietary strategies to prevent disease^22,34–36^. Yet, no studies have examined how genetic variability that already exists in common bean genotypes impacts the composition and function of the human gut microbiome.

Common bean varieties consumed today exhibit a distinct, hierarchical population structure resulting from two independent centers of domestication, one in Central America (Mesoamerican gene pool) and a second in South America (Andean gene pool). Common bean varieties descending from these two initial gene pools show distinct ecological characteristics and geographic ranges, haplotypes, and allele frequencies and are called eco-geographic landraces^37^. The Durango, Jalisco, Mesoamerica, and Guatemala landraces were domesticated from the Mesoamerican gene pool. While the Chile, Nueva Granada, and Peru landraces were domesticated from the Andean gene pool. In this study, we focused on the Middle American Diversity Panel (MDP), which comprises inbred lines representing genetic diversity across the Durango and Mesoamerican landraces of the Mesoamerican gene pool. The MDP includes six market classes defined by bean size, shape, and color. Navy and black bean market classes were derived from the Mesoamerican landrace, while the pink, small red, pinto, and great northern market classes were derived from the Durango landrace. Compositional genome analyses suggest only modest levels of introgression and hybridization between the market classes, which likely reflects the historic emphasis on maintaining bean size, shape and color phenotypes within the market classes^38^.

We recently reported the use of in vitro fermentation reactions to study the effects of naturally occurring genetic variation in sorghum^39–41^ as well as seed protein composition in maize^42^ on the human gut microbiome. We miniaturized and automated the in vitro digestion and microbiome fermentation (Automated in vitro Microbiome Screening—AiMS) used for phenotyping, which allowed us to conduct genetic analysis using the AiMS fermentations patterns as traits. In the current study, AiMS phenotyping was used with a comprehensive genome-wide association (GWA) approach to link genetic variants segregating in common beans to changes in the structure and function of multiple human gut microbiomes during fermentation. Our results show the population structure of common beans explains a significant proportion of the variation in microbiome fermentation patterns. GWA models incorporating controls for population structure further identified Multiple Effect Loci (MEL) that enhance or suppress the effects of variation associated with population structure. We validated the allelic effects of a single MEL across a larger panel of human gut microbiomes and further used molecular complementation for this MEL to demonstrate the observed microbiome phenotypes are likely driven by variation affecting the biosynthesis of one or more saponins.

## Results

### Effect of population structure of common bean on human gut microbiome composition and function

**Fig. 1:**
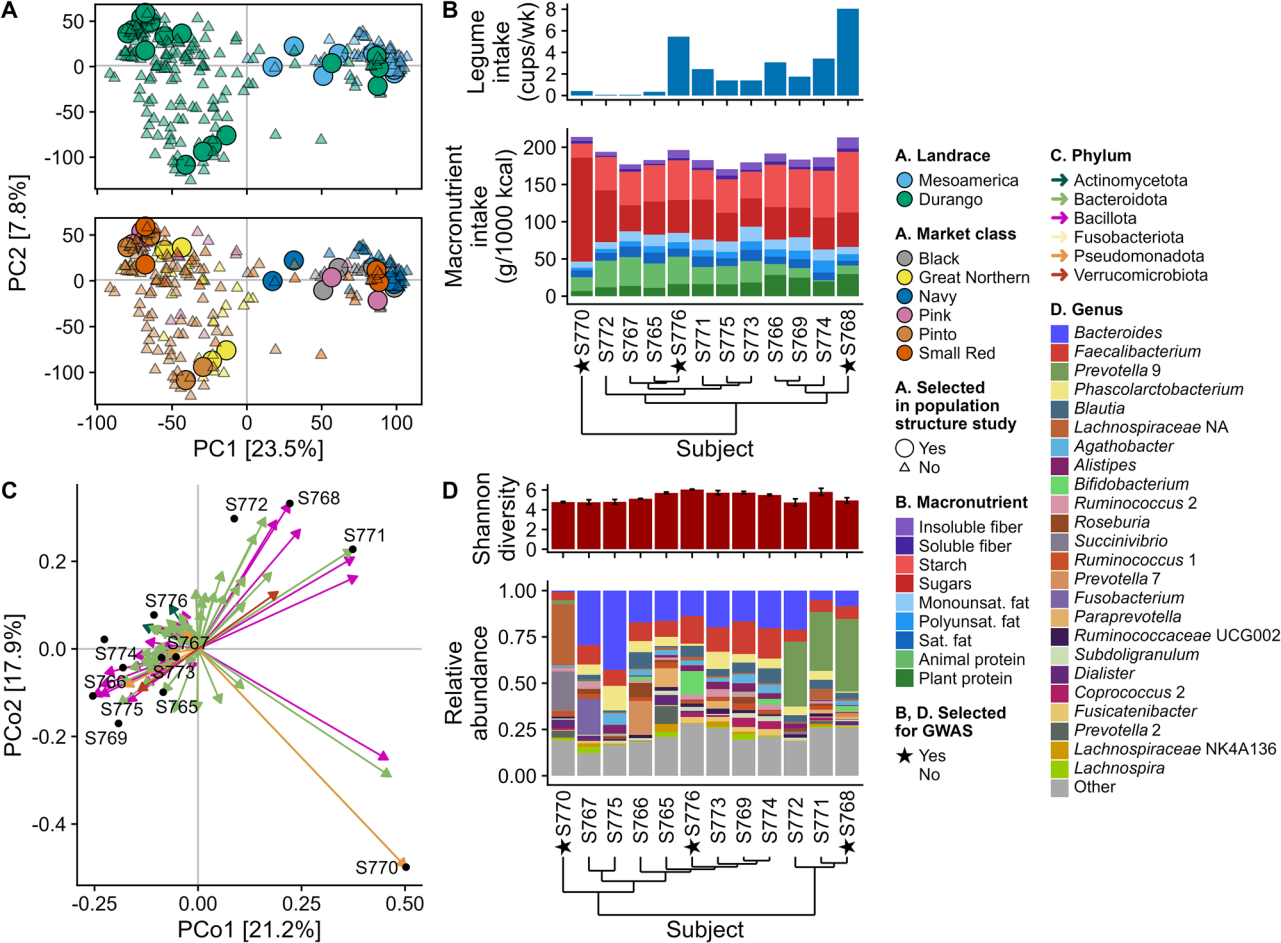
Genetic variability in common bean genotypes in the Middle America Diversity (MDP) panel, dietary intake of fecal donors, and composition of the fecal microbiota used in this study. **A** Principle component (PC) analysis biplot of the single nucleotide polymorphism (SNP) marker data from the MDP by landrace and market class with genotypes selected in the population structure study indicated; **B** habitual legume and macronutrient intake of fecal donors as reported in the Dietary History Questionnaire III; **C** principal coordinates (PCo) analysis biplot of fecal microbiotas based on Bray-Curtis distance with vectors for the top 100 most abundant amplicon sequence variants plotted; **D** α-diversity and abundance of dominant genera in baseline fecal samples. In panels **B** and **D**, clustering was done using Ward’s method based on Euclidean distance.

First, we examined whether gut microbiota composition and function in AiMS fermentations would vary by population structure within the MDP. Therefore, we selected four bean genotypes that represented the genetic diversity within each landrace and market class, based on single nucleotide polymorphism (SNP) marker data (Fig. 1A). The bean genotypes were milled, steamed, and subjected to in vitro digestion and dialysis before being used as substrates in an in vitro fermentation study using human fecal microbiotas from twelve donors.

The fecal donors consisted of seven males and five females ranging in age from 28-41 years (median: 28.5 years). These donors reported a wide range of legume and macronutrient intakes based on food frequency questionnaire responses (Fig. 1B). The donors clustered into three groups, where S770 was in a group by themselves with very high sugar intake, and S766, S769, S774, and S768 had generally higher legume intake and plant protein intake compared with the other microbiomes. The fecal microbiotas from these fecal donors also clustered into three groups (Fig. 1C, D): S770 was alone, with one ASV corresponding to *Succinivibrio* (Pseudomonadota) contributing to the location of this outgroup. S768, S771, and S772 had high abundances of *Prevotella*, while seven other microbiomes had higher abundances of *Bacteroides*.

**Fig. 2:**
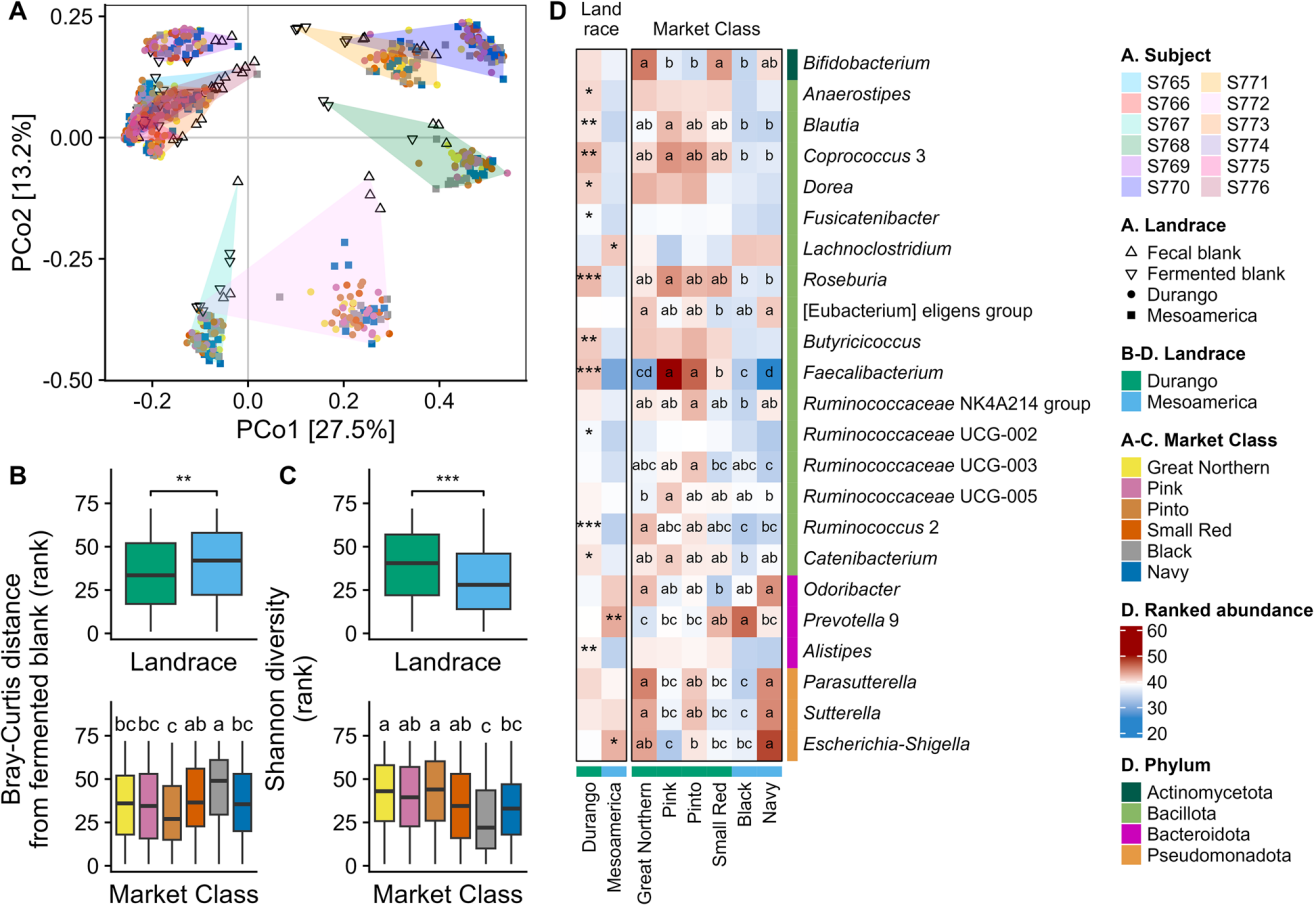
Diversity and abundance of bacterial genera after in vitro fermentation of selected genotypes from the Middle American diversity panel with 12 human microbiomes. **A** Principle coordinates (PCo) biplot based on Bray-Curtis distance among samples; **B** Bray-Curtis distance from the blank after fermentation by landrace and market class; **C** Shannon diversity of the microbiota after fermentation by landrace and market class; **D** heatmap of differential genera by landrace and market class; *p < 0.05, **p < 0.01, ***p < 0.001 indicate significant differences between landraces (Kruskal-Wallis test with Benjamini-Hochberg-adjusted *p* values); the boxplots span the 25th to the 75th percentile of the data with the center line representing the 50th percentile (median) and whiskers showing extreme data that are up to 1.5*the range of the box; no points were beyond this and therefore no extreme outliers are plotted; in the heatmap, the asterisks are placed in the cell corresponding to the landrace with the higher abundance; ^abcd^ boxplots or heatmap cells marked with different letters are significantly different (Kruskal-Wallis test with Benjamini-Hochberg-adjusted *p* values followed by Dunn’s test to identify significant differences among market classes, *p* < 0.05); *N* = 900 (12 microbiomes X (24 bean genotypes + 1 control) X 3 replicates).

Both landrace and market class had significant effects on microbiome-wide ecological diversity metrics after in vitro fermentation across all 12 subjects (Fig. 2A; Supplementary Table S1). β-diversity metrics of individual microbiomes showed significant microbiome-wide differences driven by landrace in six microbiomes while market class drove significant differences in seven microbiomes (Supplementary Table S1). Specifically, lines from the Mesoamerica landrace—particularly from the black bean market class—shifted the microbiota composition to a greater degree than Durango lines relative to a blank (Fig. 2B; Supplementary Table S2). Landrace and market class also drove significant differences in α-diversity, with lines from the Durango class resulting in higher Shannon diversity after fermentation than those from Mesoamerica (Fig. 2C; Supplementary Table S2). Great northern and pinto bean market classes maintained particularly high diversity relative to other market classes across all microbiomes (Fig. 2C; Supplementary Table S3).

At the taxonomic level, 23 genera showed significant differences among landrace or market class (Fig. 2D, Supplementary Tables S4 and S5). Most of the responsive genera belonged to two butyrate-producing families: *Lachnospiraceae* (*Anaerostipes, Blautia, Coprococcus* 3*, Dorea, Fusicatenibacter*, *Lachnoclostridium*, *Roseburia*, and [Eubacterium] eligens group) and *Ruminococcaceae* (*Faecalibacterium*, *Ruminococcaceae* NK4A214 group, *Ruminococcaceae* UCG-002, *Ruminococcaceae* UCG-003, *Ruminococcaceae* UCG-005, and *Ruminococcus* 2). A single genus of the *Ruminococcaceae* family, *Faecalibacterium*, accounted for the greatest microbiome-wide effects between landraces and among market classes, with higher abundances in Durango lines—particularly pink and pinto—compared with Mesoamerica lines.

### AiMS phenotyping of the common bean MDP

**Fig. 3:**
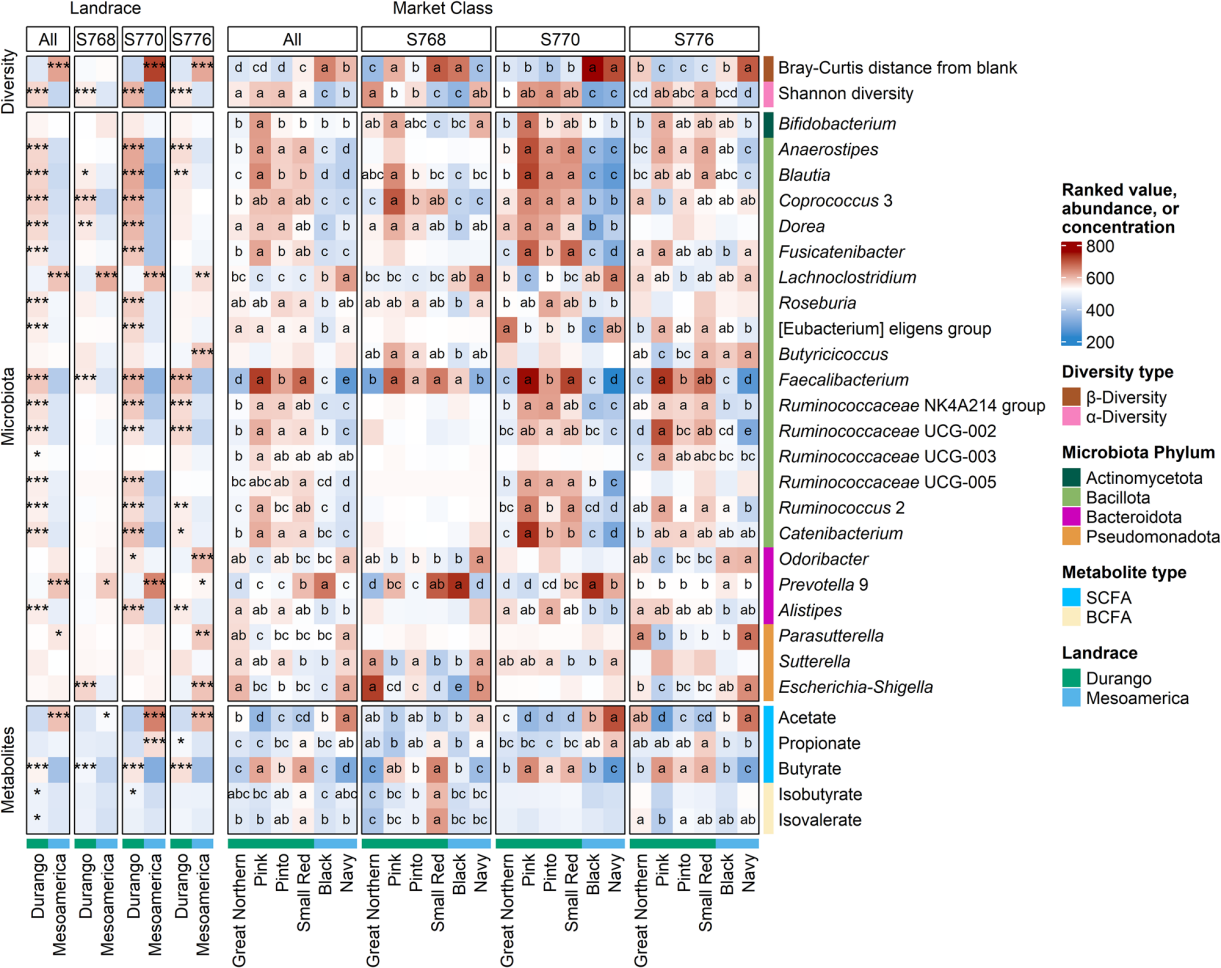
Diversity and abundance of bacterial genera and metabolites after in vitro fermentation of 299 genotypes from the Middle American diversity panel with three human microbiomes. Bray-Curtis distance from the blank indicates the distance from the blank after fermentation; for microbiota composition, all 23 genera that were significant in the population structure study (Fig. 2) are shown (56 other genera showed significant differences and can be found in Supplementary Tables S9 and S10); “All” panels refer to the average across individual microbiotas (S768, S770, and S776); heatmap cells marked with asterisks indicate this landrace was significantly higher than the other landrace within panel and row (Kruskal-Wallis test with Benjamini-Hochberg-adjusted *p < 0.05, **p < 0.01, ***p < 0.001); ^abcd^ heatmap cells marked with different letters are significantly different within panel and row (Kruskal-Wallis test with Benjamini-Hochberg-adjusted *p* values followed by Dunn’s test to identify significant differences among market classes, *p* < 0.05); *N* = 3204 (*n* = 1056 per microbiome with 36 genotypes replicated seven times and the remainder plus fecal and fermented blanks replicated three times).

Next, we used the AiMS platform to phenotype 299 bean genotypes across the entire MDP panel. In this experiment, using all 12 microbiomes was not feasible; therefore, S768, S770, and S776 were selected to cover the compositional diversity as well as the fecal donor dietary diversity across the 12 microbiomes (Fig. 1B–D). The effects of landrace and market class across the entire MDP panel using the three selected microbiomes showed many of the same effects that were observed with selected bean genotypes across all 12 microbiomes. For example, lines from the Mesoamerica landrace, especially black beans, shifted the microbiota composition to a greater degree than Durango lines relative to a blank (Fig. 3; Supplementary Tables S6–S8). Durango lines also resulted in higher Shannon diversity after fermentation than those from Mesoamerica. At the taxonomic level, *Prevotella* 9 and *Lachnoclostridium*, were more abundant when treated with Mesoamerica lines across the whole MDP, whereas *Faecalibacterium*, *Blautia*, *Anaerostipes*, *Ruminococcaceae* UCG-002, *Coprococcus* 3, and *Dorea*, where elevated in reactions with lines from the Durango landrace (Fig. 3; Supplementary Table S9). *Faecalibacterium* was again the genus with the greatest differences among market classes, with higher abundances when microbiomes were treated with pink, pinto, or small red beans from the Durango market class (Fig. 3; Supplementary Table S10).

Some taxa showed remarkable consistency across microbiomes. For example, *Faecalibacterium* and *Blautia* showed higher abundances after treatment with Durango lines compared with Mesoamerica lines in all three microbiomes, while *Prevotella* 9 and *Lachnoclostridium* showed higher abundances after treatment with Mesoamerica lines (Fig. 3). Many other genera showed consistent effects across two out of the three microbiomes.

Short and branched chain fatty acids were also quantified after fermentation across the MDP panel. Acetate and butyrate showed the greatest differences across landrace and market class, with acetate concentrations elevated in microbiomes treated with Mesoamerica lines, driven more by the navy bean lines than the black bean lines, while butyrate concentrations were elevated in microbiomes treated with Durango lines, primarily due to the pink colored bean lines (Fig. 3, Supplementary Tables S11 and S12).

**Fig. 4:**
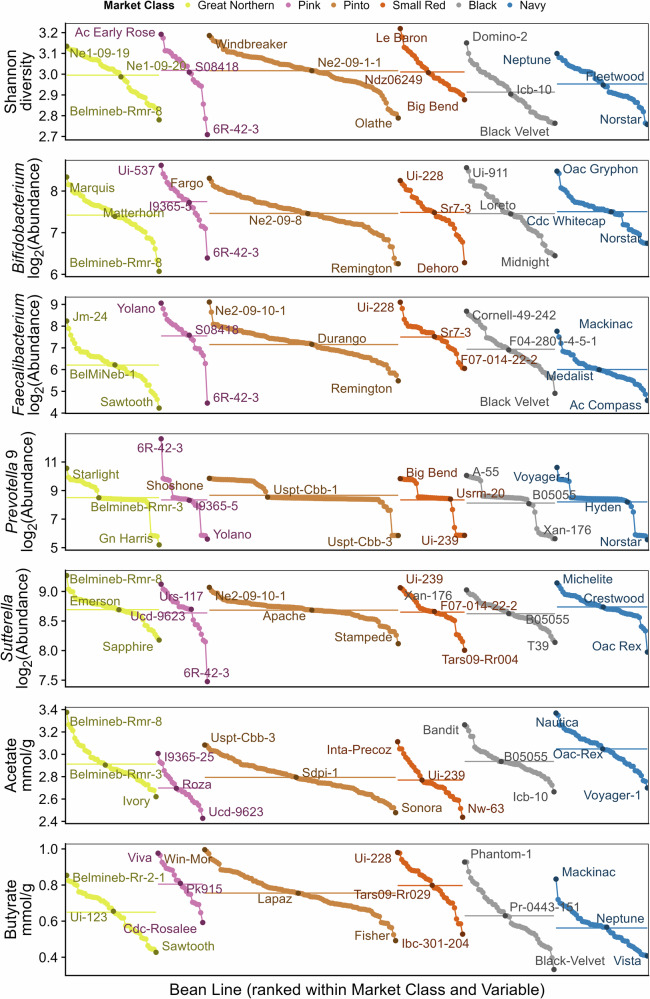
Genetic variation between and within market classes for selected microbiome variables. Average Shannon diversity, log_2_ rarefied abundances, and concentrations of selected variables from fermentations of common bean lines across the entire MDP; bean genotypes from each market class resulting in the highest and lowest value and the value closest to the market class mean for each variable are labeled; the horizontal lines within market class indicate the mean across the whole market class; significant differences among market classes are shown in the “All” columns in Fig. 3.

Surprisingly interesting observations were made when examining the variation in taxon abundances after treatment of microbiomes with different bean lines belonging to the same market class. Effects of this within-market class genetic diversity are illustrated in Fig. 4 on selected microbiome traits. These traits were selected to represent each of the major groups of variables examined [diversity, composition (one genus from each phylum), and metabolites] that showed significant differences across market classes. For some traits, including Shannon diversity, *Bifidobacterium*, *Faecalibacterium*, acetate, and butyrate, substantial variability existed within market class—almost encompassing the entire range of responses observed—with significant differences detected primarily because of the large number of lines examined within market class. In contrast, representative genera from Bacteroidota and Pseudomonadota, *Prevotella* 9 and *Sutterella*, respectively, showed almost no variability across or within market class and significant differences were primarily driven by lines at the extremes of each market class. Thus, even with the strong historical selection for seed size, shape, and color within a market class, there is still tremendous biochemical and compositional variation within market classes that can drive distinct patterns of fermentation by the microbiome.

### Identifying heritable microbiota-active traits among common bean genotypes

Next, we used AiMS based phenotypes to localize genetic variation in the MDP that can affect microbiome phenotypes beyond the population structure. Initially, 312 features were identified across the three microbiomes (148 bacterial ASVs, 92 genera, 45 families, 18 diversity metrics, and 9 SCFAs). In addition to these traits, we also generated “polymicrobial traits,” which represented collective changes in abundances of more than one trait (regardless of taxonomic relationships) during the fermentation of the common bean genotypes (see Supplemental Methods, Supplementary Table S13). The polymicrobial traits included 16 features generated from Principal Component Analysis (PCA) of the covariance/correlation matrices and eight features from Canonical Discriminant Analysis (CDA) of raw and standardized coefficients.

Due to the large numbers of microbiome traits, we calculated broad-sense heritability (H^2^) and excluded traits with H^2^ < 0.1. This resulted in 63 traits from S768 (H^2^ 0.42-0.1), 87 traits from S770 (H^2^ 0.56-0.1), and 64 traits from S776 (H^2^ 0.69-0.1) (Supplementary Tables S14 and S15). Members of *Ruminococcaceae* had the highest H^2^ values in the microbiome from S776 (genus *Faecalibacterium* 0.56, ASV6_*Faecalibacterium* 0.56, and ASV246_*Faecalibacterium* 0.49) while members of the *Prevotella* and *Phascolarctobacterium* were the most heritable in the microbiome from S768 (ASV217_*Prevotella* 0.424, ASV1_*Phascolarctobacterium* 0.374, and genus *Phascolarctobacterium* 0.369). The microbiome from S770 was intermediate with members of *Ruminococcaceae*, *Lachnospiraceae*, and *Prevotellaceae*, all having H^2^ values in the range of 0.40–0.56 (ASV5_*Prevotella* 0.56, genus *Blautia* 0.51, and genus *Lachnospira* 0.48). Importantly, many of these highly heritable taxa were the same as or highly related to taxa that showed significant effects of population structure within the MDP panel (Fig. 2).

Lastly, the heritability of an important bacterial fermentation product, butyrate, varied substantially among the three microbiomes, with values ranging from 0.44 in S776, 0.26 in S770, and 0.10 in S768 (Supplementary Table S14). The relatively higher H^2^ level in the S776 microbiome was consistent with the high degree of heritability of butyrate-producing *Faecalibacterium* in this microbiome (highest H^2^ value of 0.56) and the high degree of correlation between the abundance of this butyrate-producing organism and butyrate levels in the fermentations (R = 0.93, *p* < 0.001).

### Architecture of significant genetic associations identifies Multiple Effect Loci

**Fig. 5:**
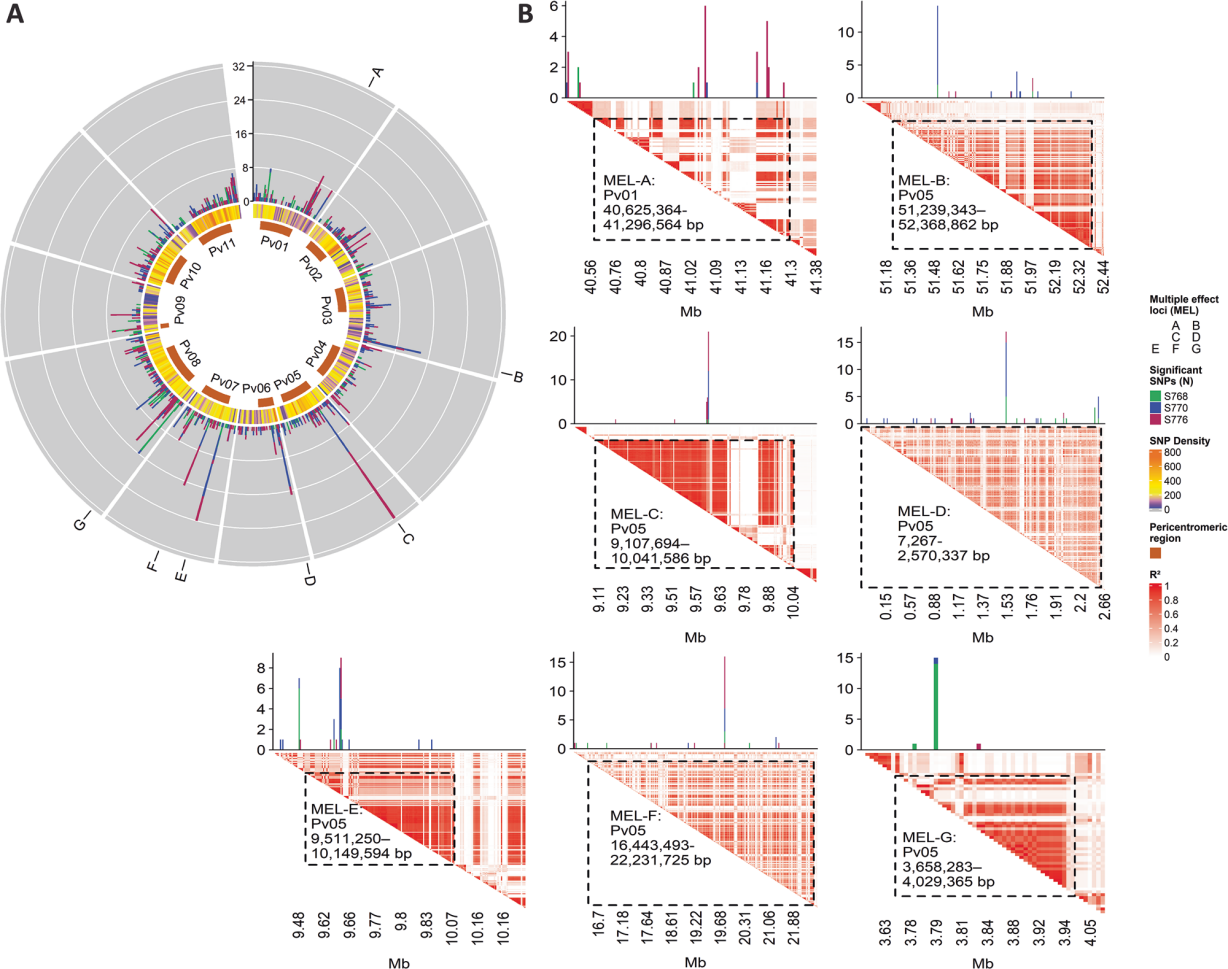
Localization of MEL on the common bean genome. **A** Circular stacked bar plot of the number of microbiome traits that were significantly associated with SNPs in the common bean genome (Bonferroni adjusted *p* value < 0.05; LOD > 6.5; SNPs were binned into 0.1 Mb bin size). MEL are marked across the genome as (**A**–**G**). The inner rings show the SNP density in each bin and the pericentromeric regions. **B** Linkage disequilibrium heatmaps surrounding the seven highly pleiotropic loci and defining boundaries of the MEL (A–G). Stacked bar plots represent the number of significant traits from each microbiome and are centered over their respective significant SNPs. The dashed boxes show the boundaries that comprise the MEL.

GWAS revealed at least one significant trait association marker for all but one of the microbiome traits with H^2^ ≥ 0.1 (*n* = 213 out of 214) (Bonferroni adjusted *p* value < 0.05; LOD > 6.5; Supplementary Table S16). Several locations in the genome contained clusters of proximal SNPs associated with >15 combined microbiome features from at least two subjects (Fig. 5A; Supplementary Table S17). These MEL were located on chromosomes *Pv*01 (MEL-A), *Pv*03 (MEL-B), *Pv*05 (MEL-C), *Pv*06 (MEL-D), *Pv*07 (MEL-E and MEL-F), and *Pv*08 (MEL-G) and are likely to have more globalized effects on the microbiome than to loci associated with fewer microbiome features. The sizes of the MEL were defined by linkage disequilibrium analysis and ranged in length from 371 kb for MEL-G to 5.7 Mb for MEL-F (Fig. 5B). In most cases, a single SNP or a small number of adjacent SNPs accounted for most GWAS signals within a given MEL. For example, MEL-C was largely defined by three SNPs within a 100-basepair region (9,615,001 bp, 9,615,026 bp, and 9,614,950 bp) and accounted for 32/34 of the significant associations in this MEL. In particular, 21 different traits were associated with a single SNP at position 9,615,026 bp on *Pv*05 in MEL-C.

Metrics of α-diversity had significant associations to multiple MEL. Shannon diversity was significantly associated with four MEL (MEL-B, MEL-D, MEL-F, MEL-G; Supplementary Table S17). Pielou’s evenness had the next highest significant associations at three to MEL-B, MEL-F, and MEL-G. MEL-G had significant associations with three metrics of α-diversity and two MEL (MEL-D, MEL-F) had significant associations to α-diversity in two microbiomes. Therefore, these may be locations of interest for genetic variation in common bean that affects the microbiome at a community level. Within individual MEL, variation in the MEL affected groups of taxonomically related organisms in at least two microbiomes, implying similar physiological effects on distinct microbiomes. For example, MEL-C affected different ASVs of the butyrate-producing families of *Lachnospiraceae* and *Ruminococcaceae* in the microbiomes of S770 and S776 (Supplementary Table S17). This similar taxonomic feature was also shared by MEL-D, which also affected members of these same families in all three microbiomes. In fact, MEL-A, MEL-B, MEL-C, and MEL-D each were associated with changes in the abundance of multiple members of the butyrate-producing family, *Lachnospiraceae*, in the microbiomes of all three donors. Similarly, MEL-C, MEL-D, MEL-E, and MEL-F had shared effects on multiple members of the butyrate-producing *Ruminococcaceae* in all three donors.

Each MEL included significant associations with one or more polymicrobial traits (Supplementary Table S17). As expected, within a given microbiome, there were many overlapping associations where the same SNP was significantly associated with the individual taxon that was also the main driver of the significant polymicrobial trait. For example, overlapping associations of individual and polymicrobial traits from S776 to MEL-C showed hits of the polymicrobial trait HypCorrPC2 (driven largely by *Faecalibacterium*, *Coprococcus* 3, *Lachnospiraceae* UCG-004) as well as the individual taxonomic traits of multiple ASVs of *Faecalibacterium* and *Lachnospiraceae*. The same pattern was also present in MEL-D, where a SNP at position 1,531,686 bp on chromosome *Pv*06 had significant associations with 17 different microbiome traits. These traits included *Faecalibacterium* (in S770 and S776 microbiomes) and several polymicrobial traits, including PCs (GenCovPC1, HypCorrPC1, HypCovPC1) and CDs, (StdCD2, StdCD4) that each included *Faecalibacterium* as one of the organisms comprising a substantial proportion of the variance. In contrast, associations of polymicrobial traits to genetic regions outside MEL contained none or only a small number of individual trait associations.

Lastly, the short chain fatty acids acetate, propionate, and butyrate were shown to be significant in multiple MEL. Acetate was significant in five MEL (MEL-B, MEL-C, MEL-D, MEL-E, MEL-F) and propionate (MEL-C) and butyrate (MEL-E) were significant in one MEL each. Not surprisingly, these SCFA had overlapping hits within MEL to SCFA-producing taxa. For example, MEL-C, which was significant for propionate, was also significant for the propionate-producing genera, *Bacteroides* and *Prevotella*, while MEL-E, which was significant for butyrate, had several genera and ASVs of butyrate-producing bacteria significant such as *Faecalibacterium, Ruminococcaceae* spp., and *Lachnospiraceae* spp. (Supplementary Table S17). Altogether, these microbiome features show that genetic variation in common bean can broadly influence a microbiome and may, therefore, represent unique opportunities for the discovery of novel microbiome-active traits.

### Genetic variation in MEL-C on *Pv*05 drives convergent microbiome responses

**Fig. 6:**
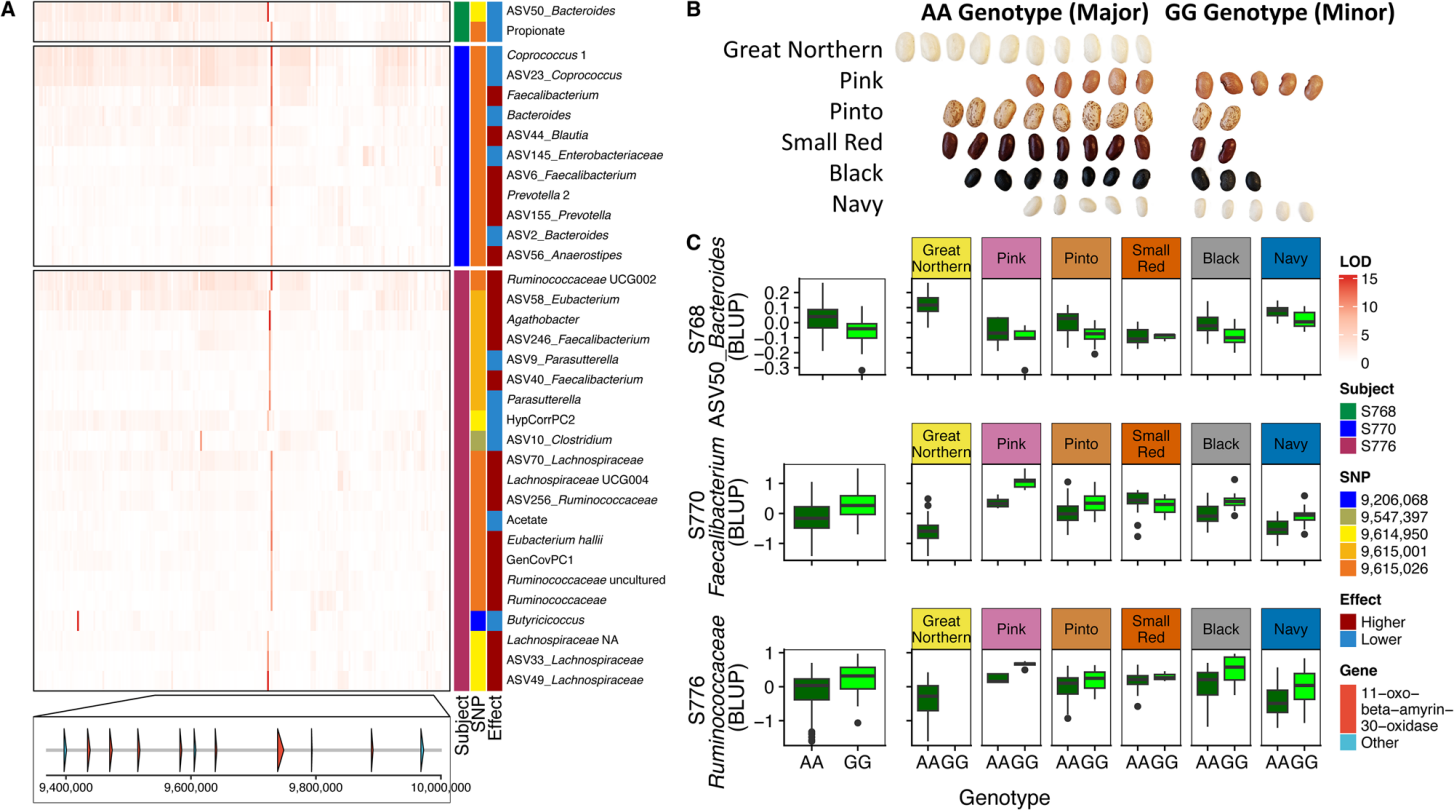
MEL-C. **A** Heatmap showing the LOD scores for microbiome features with significant marker-trait associations within MEL-C across all three subjects; red indicates that the effect of the minor allele on that trait (feature) is higher than the major allele; blue indicates that the effect on that trait is lower in lines with the minor allele than the major allele. The bottom of the heatmap shows the genomic region of *Pv*05 corresponding to genomic positions 9,400,000-10,000,000 bp in the reference genome *P. vulgaris* G19833 (v2.1). **B** Beans depicting the proportion of lines in each market class of the MDP carrying genotypes associated with the major and minor GWAS alleles; each bean represents 10% of the lines of that market class. **C** Allelic effects of major and minor alleles for three strongly affected traits at MEL-C SNP position 9,615,026 bp plotted by genotype and by genotype X market class.

To further examine relationships between genetic variation in a MEL and the microbiome phenotypes, we focused on the 933 kb region of MEL-C (Fig. 6A). Three SNPs accounted for 32 of the 34 significant associations within MEL-C, with a single SNP at *Pv*05:9,615,026 bp accounting for 21 significant associations alone, the most associations of any SNP across the genome (Supplementary Table S17). The minor G allele at *Pv*05:9,615,026 bp varied in frequency across the different market classes, being absent in great northern and low frequency in pinto, small red, and black bean classes (Fig. 6B). Allelic effects of the associated taxa from S770 showed that beans homozygous for the minor G allele at *Pv*05:9,615,026 bp exhibited an increased abundance of *Prevotella*, *Blautia* (*Lachnospiraceae*), *Anaerostipes* (*Lachnospiraceae*), and *Faecalibacterium* (*Lachnospiraceae*), and corresponding decreases in abundances of *Bacteroides* and *Coprococcus* (Fig. 6A). The microbiome from S776 showed similar responses, with taxa from *Ruminococcaceae* and *Lachnospiraceae* having higher abundances associated with the minor G allele along with the polymicrobial trait GenCov.1 (which was driven largely by [Eubacterium] hallii group) (Fig. 6A). Notably, the shared allelic effects of the G allele at *Pv*05:9,615,026 bp on members of *Ruminococcaceae* from microbiomes of S770 and S776, increased abundances of *Faecalibacterium* (S770), and increased abundances of the *Ruminococcaceae* (S776, particularly *Ruminococcaceae* UCG-002) illustrate convergent allelic effects at this locus across diverse microbiomes (Fig. 6A). Additionally, the overlapping taxonomic associations between [Eubacterium] hallii group and the polymicrobial trait GenCov.1 (driven by [Eubacterium] hallii group) further strengthen the significance of MEL-C.

The overall allelic effect of *Pv*05:9,615,026 bp on the individual microbiome traits was generally shared across market classes (as would be expected from inclusion of common bean population structure in the GWAS model) but in some instances the allelic effects were muted within one or two market classes. This combination of effects from population structure and genetic variation at a significant SNP is depicted with selected traits from each microbiome in Fig. 6C, where allelic effects for the SNP at *Pv*05:9,615,026 bp are plotted for the entire MDP and by market class. Thus, the overall effects of variation within a MEL on a given microbiome trait is a culmination of effects from population structure and allelic variation.

### Validation of allelic effects at MEL-C across 12 microbiomes

**Fig. 7:**
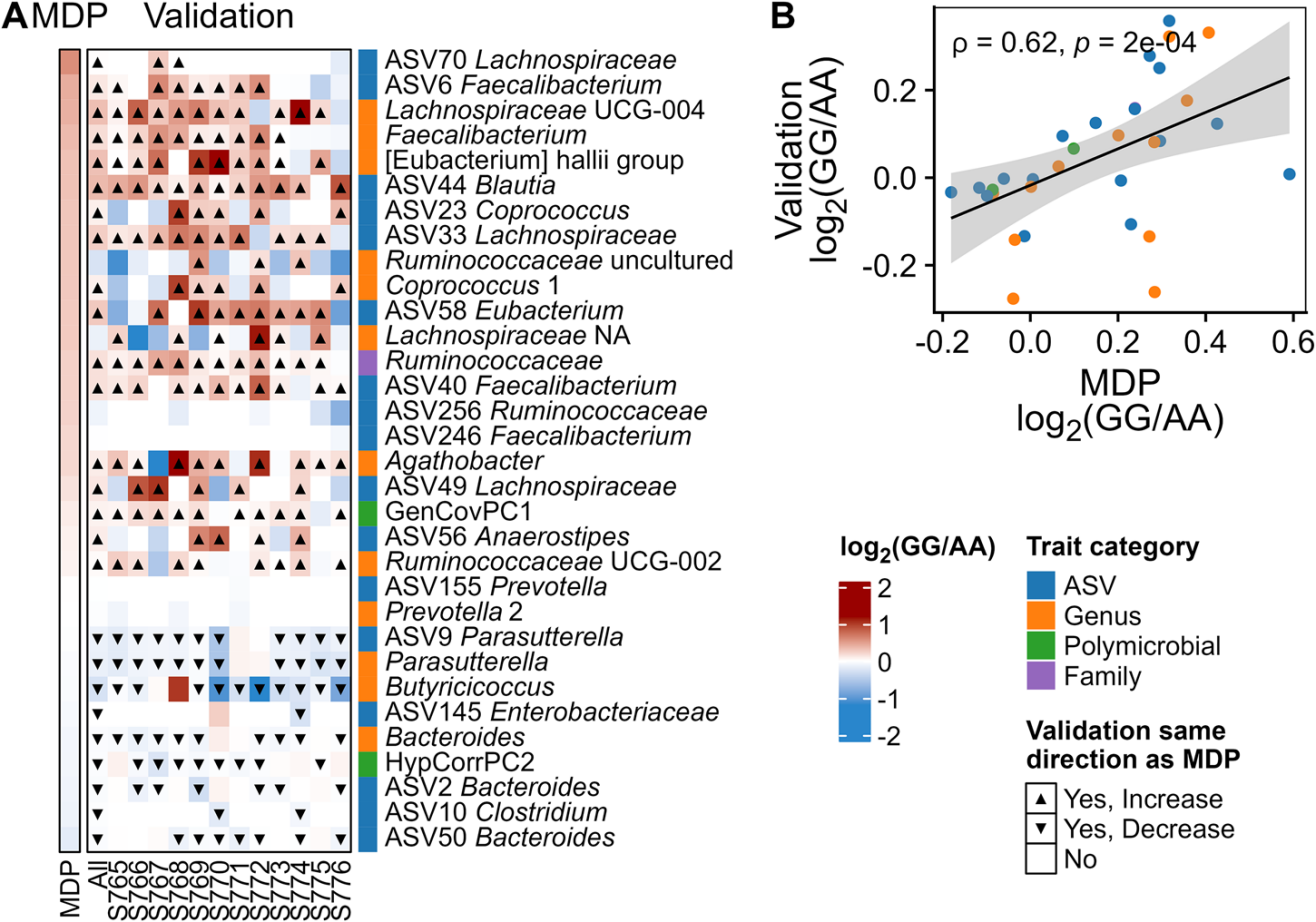
Validation of allelic effects at MEL-C. **A** Heatmap of the log_2_ fold change in relative abundance of significant microbiota traits from MEL-C from GG (minor) versus AA (major) genotypes in the Middle American Diversity Panel (MDP) and in pools of GG versus AA genotypes fermented using microbiotas from 12 subjects (Validation); “All” refers to the average across all microbiotas in the validation study; **B** scatter plot of the MDP and All columns in the heatmap in panel A; Spearman correlation coefficient and *p* value are indicated; shaded region represents the 95% confidence interval of the regression line.

Common bean lines representing each market class were pooled within market class based on their genotype the SNP marker at *Pv*05:9,615,026 bp and fermented with the original 12 microbiomes to test whether the allelic effects of MEL-C in three microbiomes would persist in additional human microbiomes. Thirty-two of the 34 microbiome traits that were significant at MEL-C in the GWAS were analyzed (i.e., all traits except SCFA). Remarkably, when averaged across the 12 microbiomes, 25/32 of the traits showed allelic responses in the same direction as the MDP panel (Fig. 7A). The similarity was also apparent in the correlation between the average log_2_ fold change observed for each taxon between beans carrying different alleles of MEL-C in the original MDP data (averaged for lines having each genotype at *Pv*05:9,615,026 bp) and the lines pooled by genotype at *Pv*05:9,615,026 bp across the 12 microbiomes (Fig. 7B). Thus, the effects of allelic variation *Pv*05:9,615,026 bp that were initially detected with three diverse microbiomes also translated across additional microbiomes in lines pooled by genotype, illustrating the repeatability of the effects of genetic variation at this locus on diverse microbiomes.

### Molecular complementation of allelic effects at MEL-C

**Fig. 8:**
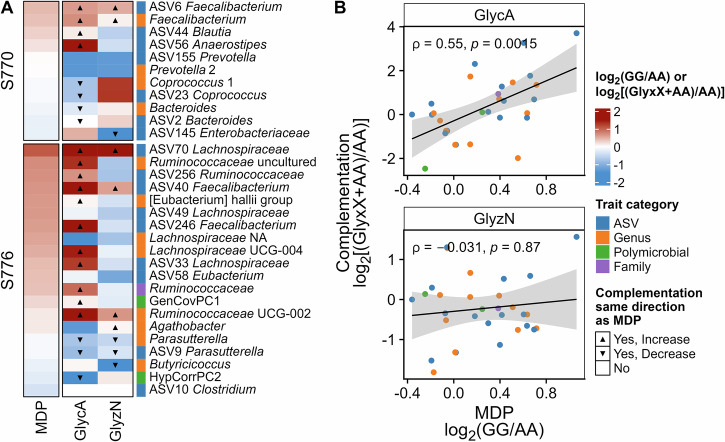
Molecular complementation with glycyrrhetinic acid (GlycA) and glycyrrhizin (GlyzN). **A** Log_2_ fold change in relative abundance of significant microbiota traits from MEL-C for microbiotas from subjects 770 and 776 (S770, S776) from GG (minor) versus AA (major) genotypes in the Middle American Diversity Panel (MDP) and in pools of seed from AA genotypes with the addition of GlycA or GlyzN (1% weight/weight of seed powder) relative to the AA genotype pools alone; **B** scatter plots of the data shown in the heatmap in panel (**A**) comparing the MDP data versus the GlycA and GlyzN-spiked samples; Spearman correlation coefficient and *p*-value are indicated; shaded region represents the 95% confidence interval of the regression line.

The SNP at *Pv*05:9,615,026 bp that accounted for most of the signal at MEL-C was located against the common bean reference genome, *P. vulgaris* G19833 (v2.1)^43^. This SNP was within a tandem array of seven genes encoding homologs of 11-oxo-β-amyrin-30-oxidase (amyrin oxidase) (Fig. 6A), a cytochrome P450-type oxidase that is involved in the biosynthesis of saponins^44^. Thus, we tested the hypothesis that variation in saponin synthesis could explain the broad effects of MEL-C on the human gut microbiome using molecular complementation with the saponin precursor, glycyrrhetinic acid (GlycA), and its glycosylated derivative, glycyrrhizin (GlyzN). GlycA or GlyzN were spiked into bean lines carrying the major genotype (AA) at 1% and subjected to in vitro fermentation to determine if these lines would mimic the effects of the minor genotype lines. Only microbiomes S770 and S776 were assayed because microbiome S768 only had two significant marker-trait associations at *Pv*05:9,615,026 bp. After fermentation, the effects of GlycA mimicked the directionality of the allelic effects of the minor allele at *Pv*05:9,615,026 bp, albeit in many cases highly amplified, for 8/11 traits that were significant in microbiome S770 and 14/20 traits that were significant in microbiome S776 (Fig. 8A). GlyzN showed some of the same allelic effects of the minor allele at *Pv*05:9,615,026 bp, but not nearly as many as GlycA. Correlation analysis corroborated the similar allelic effect in the MDP versus the complementation study with GlycA and not GlyzN (Fig. 8B).

## Discussion

Historical breeding and contemporary economic pressure to produce common beans with defined size, shape, color and organoleptic properties has led to distinctive biochemical composition across and within different populations and market classes of this crop^45–47^. Our study highlights how these efforts inadvertently converged to create IIGEs affecting the human gut microbiome and likely the gut ecosystem.

Within the context of the MDP population structure, the landraces, Durango and Mesoamerica, as well as the individual market classes within each of these landraces, induced differential effects on the AiMS fermentation patterns across diverse human microbiomes. Two abundant gut microbiota families, *Ruminococcaceae* and *Lachnospiraceae*, which contain many organisms that have been associated with positive human health outcomes^48^, were particularly affected by landrace and market class. Typically, both families were more abundant following the fermentation of beans from market classes of the Durango landrace compared with Mesoamerica.

Importantly, we also detected substantial variation in microbiome fermentation patterns across and within market classes. For example, on average, pink beans, led to the highest abundance of *Faecalibacterium*, a genus that contains beneficial species with desirable immunomodulatory characteristics that reduce susceptibility to inflammatory bowel disease^49^ and susceptibility to tumors in mouse models^50^, relative to the other market classes tested. However, even within pink beans, substantial variation was noted. For example, the pink bean cultivar (cv.) ‘Yolano’ drove high abundances of *Faecalibacterium*, while cv. ‘6R-42-3’ led to only moderate abundances of this beneficial genus, even compared with other market classes. Thus, these cultivars would be good candidates for clinical studies to determine the differential effects of pink bean consumption on the abundance of *Faecalibacterium*, especially if this leads to distinctive health outcomes. If so, there could be added commercial value and health benefits in production of cv. ‘Yolano’. It is also worth noting that this microbiome trait could be introgressed into other market classes, such as navy beans, which generally supported far lower levels of *Faecalibacterium*, to enhance the gut-health-promoting activity of navy beans.

Because our GWAS model incorporated population structure, we were able to identify loci where genetic variation affected the microbiome beyond the context of population structure. Across the common bean genome, we identified seven MEL that led to significant variation in multiple microbial taxa or metabolites across two or three human gut microbiomes. These regions are most likely to contain genes involved in the production of microbiota-active biochemical components within the beans.

To demonstrate how our genetic approach may be used to identify which components of common beans may cause significant impacts on the microbiome, we further examined one MEL, MEL-C. The allelic effects of this MEL on microbiome traits were validated in multiple additional microbiomes using common bean lines pooled by allele. MEL-C contained one SNP, *Pv*05:9,615,026 bp, with the most marker-trait associations. The SNP was adjacent to a tandem array of seven genes encoding a key enzyme in saponin biosynthesis. Saponins are triterpenoid plant metabolites that have a wide range of biological activities^51^. As a dietary component, saponins are noted for their beneficial effects of enhancing immunity, decreasing blood lipids, reducing cancer risks, and reducing blood glucose responses^18^. Saponins have also been reported to have inhibitory effects on bacterial species, most often noted for pathogenic microbes such as *Staphylococcus aureus* and pathogenic lineages of *Escherichia coli*^52^.

Variation in the type and abundance of saponins has not been systematically studied in *P. vulgaris* L., but these molecules are known to be most concentrated in the seed coat, and in small-scale studies, substantial variation in saponin concentration has been reported between lines^53,54^. For example, saponin characterization from different lines of black beans have shown a similar array of group A and group B saponins in the seed coat, primarily soyasaponin Af, soyasaponin ag, and soyasaponin bg, whereas the profile from a single line of pinto beans showed soyasaponin A2, soyasaponin I, soyasaponin V, soyasaponin bg, and soyasaponin gg^55^.

Although we did not know the saponin concentration or composition of the common beans in our study, we were able to demonstrate the importance of saponins on the gut microbiome response at MEL-C using a molecular complementation approach. We showed that genotypes homozygous for the major allele at *Pv*05:9,615,026 bp mimicked the directionality of the allelic effects of the minor allele after spiking with a model sapogenin (GlycA). In contrast, spiking with the model saponin, GlyzN, did not mimic the directionality of the allelic effects of the minor allele. Although spiking genotypes homozygous for the major allele at *Pv*05:9,615,026 bp with GlycA did mimic the directionality of the allelic effects of the minor allele, in many cases the effects were highly amplified, suggesting that we may have spiked with excessive GlycA. We spiked the genotypes with the GlycA at 1% of the bean weight, which was based on Shimelis and Rakshit^56^, who reported total saponin concentrations of 0.1–1% in processed beans. However, other reports show values ranging from 0.02 to 4%^57^. Thus, our approach makes a strong case for examining the effects of saponin concentration and composition on the response of the gut microbiome to beans, with the concentration of the core sapogenin most likely responsible for the microbiome effects rather than the attached sugar moieties.

Importantly, our approach incorporated milling and cooking (steaming) steps before in vitro fermentation of the bean genotypes. Cooking beans is critical to remove antinutritional factors, such as trypsin inhibitors and phytic acid, and increase digestibility of protein and starch, prior to digestion and dialysis. However, the milling and cooking procedure that we employed was somewhat different from how beans are normally prepared. The steps of soaking and boiling in excess water, which are more typical, have been shown to have negative impacts on the concentrations of saponins and many other biochemical compounds that likely impact the gut microbiota^58^. For example, in a review of more than 15 studies on the effect of processing on saponin concentrations in beans, the soaking step was reported to reduce saponin concentrations by about 5-20%, depending on soaking time and water hardness, and then boiling the beans further decreased the saponin concentration by an additional 15-25%^57^. Most studies attribute these losses to the heat lability of saponins^30,57,59^; however, processing methods such as extrusion and baking, which involve extensive heating, result in little to no saponin degradation^60^. Therefore, the losses experienced during soaking and boiling are more likely due to leaching into the soaking or boiling water rather than true degradation. In our study, the cooking method employed did not involve filtering or removing any soaking water; however, after the digestion protocol there was a dialysis step that could have resulted in loss of saponins. Nevertheless, despite any losses experienced during dialysis, presumably enough saponins were maintained in the bean digesta for us to detect the strong microbiome signal at MEL-C.

While saponins may be more heat stable than many reports indicate, heating can change the saponin composition. For example, one class of saponins contain 2,3-dihydro-2,5-dihydroxy-6-methyl-4H-pyran-4-one (DDMP) ether-linked to a sapogenol B backbone. Heating can remove the DDMP moiety through an elimination reaction, leading to a decrease in DDMP saponins and an increase in type B saponins^61^, which would likely affect the types of microorganisms that can metabolize this compound.

More importantly for our study, saponins can also lose their sugar moieties during cooking^62^. This structural change would likely have a strong effect on microbiome traits that were associated with MEL-C, since we found that our model sapogenin mimicked allelic effects at this MEL while the model saponin did not. This emphasizes the importance of cooking on the microbiome response to beans. Future studies examining the influence of processing on saponin composition in beans and how this affects the gut microbiota composition would be appropriate.

Although our study identified several genetic regions in the common bean genome that likely affect the gut microbiome and provided one example of how our genetic approach may be used to identify specific chemical compounds of beans that may cause significant impacts on the microbiome, our study is not without limitations. First, our study used an in vitro approach to examine the effects of bean genotypes on the gut microbiome. In vitro tests lack many processes that are present in vivo, such as nutrient absorption offer an affordable way for scalability of hypothesis testing and studying the direct effects of dietary components without confounding effects of other dietary components^63^. Future studies should examine the effects identified in this study in in vivo models and in human feeding trials.

Our in vitro test also used a single timepoint after fermentation for phenotypic analysis. The use of multiple timepoints throughout fermentation may help confirm or describe new MEL. Moreover, to validate these microbiome-trait associations and obtain a more complete picture of the genetic architecture in common bean, follow up studies using specialized common bean populations and in vivo feeding studies need to be done.

Apart from the limitations to the in vitro methodology, we also used relative abundance data for our phenotyping. The nature of relative abundance microbiome data generated from our AiMS phenotyping precludes us from determining whether significant allelic effects are a consequence of growth stimulation of certain microbes or inhibition of other microbes. Quantitative techniques such as plating and qPCR will be needed in the future to identify absolute abundances of target microbes of interest in response to the bean genotypes.

Finally, there were clear differences in the GWAS association patterns across microbiomes. While this was not unexpected, it illustrates that no one microbiome is likely to be an effective proxy for identifying all genomic intervals in food plant genomes. However, using the threshold of multiple effects (multiple microbes) on two or three microbiome donors for the definition of MEL, led to identification of loci where variation has potentially broad effects across diverse human microbiomes.

This demonstration of localizing genetic variation in common bean that has significant effects on the human gut microbiome creates a new strategy to capitalize on IIGEs and enhance human health through breeding of common beans. We showed not only that population structure has a strong influence on how the gut microbiome responds to beans, but substantial variation could be identified within landrace or even market class of beans. Thus, certain cultivars identified as having desirable effects on the gut microbiota could be used in breeding programs to improve the impact of poorer, but agronomically adapted, lines on the gut microbiota. In this study, we also identified saponins as a potential class of compounds that vary in beans and have strong impacts on gut microbes that have been identified as being beneficial to human health. Future studies should examine the effects of varying concentration and composition of these compounds impacts the human gut microbiota with consequences for human health.

## Materials and methods

### Middle American diversity panel

Common beans were grown at the University of Nebraska Panhandle Research and Extension Center in Scottsbluff, NE, at the Mitchell Ag Lab (41° 56.6′ N, 103° 41.9′ W, 1240 m elevation). A total of 299 common bean genotypes representing the Middle American gene pool^64^ were obtained from a single growing season (Middle American Diversity Panel; MDP; Supplementary Table S18). The MDP cultivars comprised 198 genotypes from the Durango gene pool and 101 genotypes from the Mesoamerican gene pool. Each plot consisted of two 7.6 m rows spaced 0.60 m apart. The plots received 390 mm of water (irrigation + precipitation). Genotype data for the MDP was generated from genotyping by sequencing using single reads mapped against the G19883 reference genome^65^.

### Selection of human microbiomes

Fecal samples were collected from 12 adult participants who were at least 19 years old with no known gastrointestinal disease, had not taken antibiotics in the last six months, and were not routine users of probiotic or prebiotic supplements. Collection of fecal samples from participants followed approved study protocols by the University of Nebraska-Lincoln’s Institutional Review Board (Approval Number: 20160816311EP). Each participant provided written informed consent before performing any study protocols. Additionally, all participants completed the online Diet History Questionnaire III (DHQ III) with questions about dietary recall in the past year with portion size^66^.

One fecal sample was collected from each subject using a commode specimen collection device (Fisher Scientific, Waltham, MA) and was processed under anaerobic conditions (5% H_2_, 5% CO_2_, and 90% N_2_) in the laboratory within two hours of collection. Samples were first diluted in phosphate-buffered saline containing 10% glycerol and homogenized using the BagMixer^®^ 400 CC^®^ and FILTRA-BAG® blender bags to remove large particulates. Filtered fecal homogenates were aliquoted and immediately preserved at −80 °C until the time of fermentation.

### Automated in vitro microbiome screening for microbiome phenotyping

To simulate human digestion and study genetic variation that impacts fermentation patterns by the human colonic microbiome, all common beans were subjected to the miniaturized, high-throughput Automated in vitro Microbiome Screening (AiMS) platform developed in our laboratory and previously described^39^. Importantly, each common bean genotype by donor microbiome fermentation reaction was performed in three technical replicates.

Briefly, each common bean genotype was milled using the Geno/Grinder 2025 (SPEX SamplePrep, Metuchen, NJ) integrated with Thermo Scientific VALet robotic arm, and 20 mg flour from each common bean genotype was dispensed into 1 mL 96-well plates using the Chemspeed Flex PowderDose (Chemspeed Technologies, Füllinsdorf, Switzerland). Samples were hydrated with 425 μL molecular biology grade water for 15 min. Samples containing no bean samples were also prepared as blanks. Then, the plates were immediately placed on a rack situated 5 cm above boiling water inside of a closed cooking pot and steamed for 20 min.

After cooking and cooling, the gastric phase was initiated by adding 45 μL of 500 mM HCl + 10% pepsin (w/v) (Sigma-Aldrich, St. Louis, MO) and incubated at 37 °C for one hour. The small intestine digestion phase was initiated by adding 25 μl of 0.5 M sodium maleate buffer (pH = 6, containing 1 mM CaCl_2_) and 40 μl of 0.5 M NaHCO_3_ to bring the pH to 6.0-6.5. Forty microliters of 12.5% (w/v) pancreatin (Sigma-Aldrich, St. Louis, MO) in water and 4 μl amyloglucosidase (3260 U/mL; Megazyme, Bray, Ireland) was added. The plates were incubated at 37 °C for six hours.

Digested samples were transferred to a 96-well DispoDialyzer plate (MWCO 1,000; Harvard Apparatus; Holliston, MA) containing magnetic stirring discs. The plates were dialyzed in five gallons of distilled water while stirring on a stir plate (220 rpm) along with a magnetic tumble stirrer positioned vertically next to the bucket (100 rpm) so stirring would also occur in the wells. Dialysis proceeded for 72 h at 4 °C, and dH_2_O was changed every 12 h. Following dialysis, samples were transferred to a 1 mL 96-well plate containing stainless steel stirring discs.

In vitro batch fermentations were performed inside an anaerobic chamber (containing 5% H_2_, 5% CO_2_, 90% N_2_). Digested plates were thawed, and 65 μL of 10X fermentation media^39^ and 35 μL Oxyrase (Oxyrase, Inc, Mansfield, OH) was added to each well. After one hour to reduce the oxygen concentration, samples were inoculated with 65 μL of 1:10 diluted fecal slurry and incubated anaerobically at 37 °C for 16 h. After fermentation, plates were centrifuged at 4000 x *g* for 10 min at 4 °C to separate bacterial pellets (for microbiome analysis) and supernatants (for SCFA analysis). Plates were stored at –80 °C until processing. Raw fecal slurries were also saved from each subject for measurements of baseline (0-h fermentation).

### Microbiome compositional and functional analysis

DNA was extracted to assess the microbiota’s taxonomic composition. PCR products from the V4 region of the 16S rRNA gene from each sample were subjected to 2 × 250 bp sequencing on the Illumina MiSeq platform and analyzed. Using DADA2^67^, sequences were dereplicated into amplicon sequence variants (ASV), and taxonomy was assigned based on the SILVA132 database^68^. Samples were rarefied to a sampling depth of 7,197 sequences. QIIME2 and Phyloseq^69^ were used to generate tables for taxonomic rankings, including phylum, family, genus, ASV, and diversity estimates (α and β). Taxa present in less than 10% of the samples were removed.

To determine the metabolic responses of the microbiomes to common beans, SCFA (acetate, butyrate, propionate) and BCFA (iso-butyrate, iso-propionate, iso-valerate) were extracted into diethyl ether from the fermentation supernatants and analyzed by gas chromatography^70^. Briefly, 0.1 mL internal standard (22 μL of 7 mM 2-ethylbutyric in 2 M potassium hydroxide) was added to each sample in the 96-well plate. Then, 0.2 mL of the spiked fermentation supernatant was transferred to a 2 mL screw cap tube, and 0.1 mL of 9 M sulfuric acid and 0.16 g of sodium chloride was added. After vortexing, 0.2 mL of diethyl ether was added, and samples were shaken and centrifuged at 10,000 × *g* for 5 min to help separate the aqueous layer. Lastly, 1 μL of the diethyl ether phase was injected into a gas chromatography system (Clarus 580; PerkinElmer, Waltham, MA) equipped with a fused silica capillary column (Nukol 30 m x 0.25 mm inner diameter x 0.25 µm film thickness; Sigma-Aldrich, St. Louis, MO). Quantification of S/BCFA was done by calculating response factors for each analyte relative to 2-ethylbutyric acid using injections of pure standards. Results were expressed as mmol/g undigested bean.

### MDP population structure and effects on microbiomes

To test the variability in microbiota responses to MDP population structure, four genotypes from each of the six major market classes of the MDP (Durango Landrace: great northern, pinto, pink, small red; Mesoamerican Landrace: black, navy) were selected for in vitro fermentations (*n* = 24) using the microbiomes collected from all 12 human subjects. The genotypes were selected to represent variation in single nucleotide polymorphism (SNP) marker data within and between landraces and market classes. TASSEL^71^ was used to generate principal components of the MDP from SNP genotype data, and population structure was visualized using ggplot2^72^. The selected genotypes also had variations in origin and date of release (Supplementary Table S18). Additionally, control wells that did not contain any common bean flour but were inoculated with fecal slurries and underwent the in vitro fermentation process were included in each plate. Thus, this experiment generated 900 total samples (12 microbiomes X (24 bean genotypes + 1 control) X 3 replicates). All genotypes and technical replicates per microbiomes were randomized across the wells of one individual 96-well plates (one microbiome/plate).

### GWAS experimental design

Three microbiomes were selected based on their diverse baseline taxonomic composition and differential fermentation patterns across the subset of the MDP described above. These three microbiomes were then used in AiMS fermentations to test the effects of all 299 common bean genotypes of the MDP, with each bean line x microbiome reaction being done in triplicate. The common bean genotypes were randomized in an augmented partially replicated (p-rep) design. Overall, all genotypes were randomized to wells across 4 × 96-well plates/design replicate in an incomplete block design and replicated a minimum of three times for each microbiome used in the GWAS (12 plates/microbiome). Each block of four plates contained fermentations from one microbiome. Twelve genotypes were randomly selected (without replacement) and partially replicated for each of the design replicates. Selected genotypes were assigned to wells within a plate using a partially replicated α-lattice design, arranged in a 3 × 4 rectangle across rows and columns within and across plates, within a block of four plates^73^. Out of the total 299 genotypes, across the three microbiomes, 36 were replicated seven times and the remaining 263 were replicated 3 times. Control wells that did not contain any common bean flour but were inoculated with stool microbiomes and underwent the in vitro process were included in each plate.

### Phenotypic analysis of microbiome traits

Microbiome phenotypes were defined from the taxonomic and metabolic-based output from the AiMS platform and were treated as “traits” associated with the common bean genotypes. To prioritize those individual taxa that would yield reproducible results, we filtered and used taxonomic traits (ASVs, genera, families) that were set to a threshold of a 0.15% relative abundance across all samples by subject and then normalized by log2 transformations. The transformed abundances of the individual taxa were used directly as traits in the GWAS.

Alpha-diversity metrics (Chao1, Faith’s Phylogenetic Diversity, Observed ASVs, Pielou’s Evenness, Shannon, Simpson) were calculated for each fermentation reaction using the QIIME2 platform as described above. Individual SCFA concentrations (acetate, butyrate, propionate; mmol/g bean sample) from each sample were used directly as traits in the GWAS analysis.

Polymicrobial traits were created for each subject to be used in addition to univariate taxon abundances in the GWAS. For each microbiome, only the most abundant genera that had an average sum abundance accounting for at least 90% of the overall abundance across all fermentations was used. There were nine genera in S768, 17 genera in S770 and 15 genera in S776 that were used to define the polymicrobial traits. The relative abundances of each genus were transformed using a centered log-ratio (CLR) transformation. MANOVA was performed on the transformed variables to evaluate the variability between the genera and the fixed effects of common bean genotype, in vitro digestion batch, and the plate (within each of the three digestion batches). The analysis was fit using the PROC GLM procedure within SAS 9.4 (SAS Institute Inc., 2015).

Output from the MANOVA included: (1) the genotype hypothesis sums of squares and cross-products (SSCP) matrix, (2) the error SSCP describing the variance in the genus abundances after accounting for the fixed effects and (3) a canonical discriminant analysis (CDA). Briefly, the purpose of CDA is to find a few linear combinations across the taxa (canonical components) that separate the genotypes by maximizing among genetic sums of squares of the components to the within sums of squares of the components^74^. The genotypic (hypothesis) covariance matrices were calculated by taking the SSCP matrices and dividing by their corresponding degrees of freedom and correlation matrices were calculated directly from their corresponding covariance matrices. In addition, the genetic covariance matrix, which is the multivariate analogy to the univariate broad sense genetic variance, was computed by substituting the observed genotype hypothesis covariance matrix for the expected mean squares and cross products matrix for genotype hypothesis covariance matrix and solving for the genetic covariance matrix.

Principal component analyses (PCA) for the polymicrobial traits were conducted in R Statistical Software^75^ for each of the genotype and genetic covariance matrices and their respective correlation matrices. CDA and the canonical scores were based on both the genetic hypothesis covariance and correlation matrices. The loadings from all models (both PCA/CDA loadings) were then used as weights in linear combinations of genera, creating the corresponding scores for each observation in the data set. Scores were calculated by multiplying the vector of loadings (components) by the centered (or centered and scaled) CLR abundance values. Centered data was used to calculate scores for the covariances, and the centered and scaled CLR-abundances were used for the correlations. Canonical discriminant loadings (raw and standardized canonical coefficients) came directly out of the MANOVA, and score variables were directly calculated from these. Both PCA and CDA resulted in total loading and score vectors, where is the total number of taxa included in the initial MANOVA. The first vector in each is the linear combination of taxa that accounts for the most variability overall in the input data. Only the top four components were used to calculate score variables for each of the analysis types and data combinations. There was a total of 24 polymicrobial microbiome score variables with values for each observation coming from the 16 total PCA components (4 components for each of 4 covariance/correlation data sets) and 8 total CDA components (4 components from each of the raw and standardized coefficients) as described above. The six polymicrobial methods used in the GWAS were termed: hypothesis covariance, hypothesis correlation, genetic covariance, genetic correlation, raw CDA, and standardized CDA.

### GWAS of phenotypes from AiMS

Principle components (PCs) and the centered kinship matrix were calculated in TASSEL v5.2.69 for use in GWAS^71^. Best linear unbiased predictors (BLUPs) were calculated for each microbiome trait using mixed model equations in R Statistical Software within the sommer package v4.1.1^76^. The first three PCs of the SNP data were used as fixed effects, the kinship matrix as a covariate, and the random effects, which were common bean genotype, in vitro digestion batch, plate, row within the plate and column within the plate.

For the genetic associations, SNP data for the common bean genotypes of the MDP was obtained from previously published data^65^. GWAS was performed using SNPs with minor allele frequency >0.05, which resulted in 132,314 SNPs being used. For each microbiome trait, GWAS was conducted by subject using the BLUPs for each phenotype.

A conservative estimate of broad sense heritability (H^2^) was calculated by dividing the variance explained by common bean genotype by the variance explained by genotype + all other effects, including the residual error. Separate GWAS analyses were conducted for each microbiome feature passing a heritability filter of H^2^ > 0.1 threshold. Based on this cutoff, 170 traits were removed, leaving 215 total microbiome traits. GWAS was performed using the iterating Fixed and Random Model Circulating Probability Unification (FarmCPU) algorithm^77^ as implemented within the rMVP package (v1.0.4)^78^; in R using the first five PCs calculated from the genetic marker data as covariates. Association results for SNPs from each GWAS were compiled, and Manhattan plots were generated for each trait using ggplot2 in R^72^. The significance threshold for SNPs from the FarmCPU output was set using a strict Bonferroni correction (*p* value < 3.7 × 10^–7^).

### Defining the borders of multiple effect loci

MEL were initially defined using a binning approach where each bin comprised 0.1 Mb. Bins having significant SNPs for at least 5 AiMS traits from at least two of the donor microbiomes were of interest. To further define boundaries of the MEL, linkage disequilibrium (LD) was calculated (as R^2^) in regions encompassing multiple significant SNPs identified in the GWAS. LD was used to determine the strength of LD surrounding bins with high pleiotropy to define MEL. MEL boundaries were defined as having R^2^ ≥ 0.75 regardless of the distances between SNPs.

### Validation of allelic effects at MEL-C

Due to a single SNP in MEL-C having the most significant marker-trait associations among all the SNPs studied and all three subjects having microbiome traits significantly associated in this region, this MEL was chosen for further experimental validation studies. Common bean genotypes were grouped by market class and then by the SNP marker *Pv*05:9,615,026 bp (AA genotype/major or GG genotype/minor) at the GWAS peak for MEL-C. The market class by SNP pools were created by mixing one gram of milled powder of each line into the pool (Black AA: 31 lines; Black GG: 10 lines; Navy AA: 23 lines; Navy GG: 19 lines; Pink AA: 5 lines; Pink GG: 6 lines; Pinto AA: 67 lines; Pinto GG: 15 lines) (Supplementary Table S19). Pools were not created for great northern or small red beans due to the lack of cultivars carrying the minor allele. After mixing thoroughly, a 2.5-gram sample of powder from each of the pools was digested and dialyzed as previously described^40^. After dialysis and lyophilization, the remaining solids were resuspended in 30 mL of water, and 0.25 mL of each resuspended pool was used as substrate for in vitro fermentations across the same 12 human microbiomes in triplicate (three wells for each pool X subject combination). Thirty-two (out of 34) traits that were significant at MEL-C were quantified in the AA and GG genotype pools and compared with the unpooled genotypes from the MDP data. Two traits, corresponding to SCFA, were not quantified in the validation study.

Jbrowse on Phytozome v13^79^ was used to gain insights into candidate genes and pathways associated with significant SNPs underlying the MEL using the common bean reference genome *P. vulgaris* G19833 (v2.1^43^). Genes were considered candidates if they contained a significant SNP or if there was a significant SNP within the immediate genomic region (upstream or downstream 50 kb).

### Molecular complementation with GlycA and GlyzN saponins

Pure glycyrrhetinic acid (GlycA) (18-beta-glycyrrhetinic acid; CAS: 471-53-4) or glycyrrhizin (GlyzN) (CAS: 1405-86-3) were introduced at 1% weight/weight of seed powder into the fermentation reactions (after digestion and dialysis) of each market class pool made with AA genotype. The amount of GlycA and GlyzN were determined based on reported levels of saponin found in beans^56^. AiMS reactions containing the AA genotype pools alone and AA genotype pools supplemented with GlycA or GlyzN at different levels were each inoculated individually with fecal microbiomes from S770 and S776 and used in in vitro fermentations with three replicates of genotype pool X microbiome as described above. The log2 fold-change in each significant trait (excluding SCFA, which were not quantified in the complementation study) with GlycA or GlyzN spiked into AA genotypes relative to AA genotypes alone was calculated. These data were compared with the log2 fold-changes between GG and AA genotypes in the MDP.

### Statistics and reproducibility

All statistical analyses of the 16S rRNA sequencing and S/BCFA were performed in R (v4.3.0^75^;) and R-Studio (2023.09.0 Build 463). To compare β-Diversity after in vitro fermentation, permutational multivariate analysis of variance (Adonis PERMANOVA; 999 permutations) based on Bray-Curtis distance was conducted using the vegan package (v2.6.8^80^). Differences among landrace and market class for α-diversity, microbiome taxonomic abundances, and SCFA concentrations were analyzed using Kruskal-Wallis tests with p-values adjusted using the Benjamini-Hochberg procedure using the ‘rstatix’ R-package^81^. For variables that showed a significant Kruskal-Wallis test for market class, Dunn’s test was used to determine differences among market classes, also using ‘rstatix’. For plots, data were ranked by subject (data for each subject were ranked independently) to reflect statistical comparisons. Spearman correlations were used to determine correlations between bacterial taxa and metabolites using the ‘Hmisc’ package in R^82^. Linkage disequilibrium was calculated using the ‘genetics’ package^83^. Plots were constructed using ^70^the following R-packages: ‘ggplot2’^72^, ‘ggh4x’^84^, ‘ggrepel’^85^, ‘ggtext’^86^, ‘multcompView’^87^, ‘ComplexHeatmap’^88^, and ‘circlize’^89^.

### Ethics statement

The studies involving humans were approved by the University of Nebraska-Lincoln Institutional Review Board (approval number 20160816311EP). The studies were conducted in accordance with the local legislation and institutional requirements. The participants provided their written informed consent to participate in this study.

### Reporting summary

Further information on research design is available in the Nature Portfolio Reporting Summary linked to this article.

## Supplementary information

Description of Supplementary Data

Supplementary Data

Reporting Summary

## Data Availability

Raw sequence reads from 16S rRNA gene sequencing are available in the Sequence. Read Archive under accession number PRJNA1062343^90^. The processed datasets generated during the current study are available in the figshare repository at: 10.6084/m9.figshare.30138733.v1^91^. The source data used to generate the figures can also be found in the figshare repository at: 10.6084/m9.figshare.30138844.v1^92^.

## Code availability

The processed datasets analyzed and R code generated during the current study are available in the figshare repository at: 10.6084/m9.figshare.30138733.v1^91^.
