# Supplementary material for: Genetic variation and historical breeding patterns in common bean (Phaseolus vulgaris L.) affect fermentation patterns by the human gut microbiome
Source: Commun Biol. 2025 Nov 26;8:1690. doi: 10.1038/s42003-025-09089-2 (PMC12657933; doi:10.1038/s42003-025-09089-2)
Supplement: Supplementary file 1 — Description of Supplementary Data [file 42003_2025_9089_MOESM1_ESM.docx]

**Description of Supplementary Data**

**Filename:** MDP Supplemental Tables S1-S19.xlsx

**Description**

**Supplementary Table S1. Effect of landrace and market class on global microbiota composition in terms of Bray-Curtis distance in the population structure study.** Data represent triplicate fermentations of 24 bean genotypes [four selected from six major market classes] with 12 microbiotas; permutational multivariate analysis of variance using distance matrices (PERMANOVA) for landrace and market class by microbiota (subject) and across all subjects.

**Supplementary Table S2. Effect of landrace on diversity metrics in terms of Bray-Curtis distance from the fermented blank and Shannon diversity in the population structure study.** Data represent means and standard errors by subject (microbiota) and across all subjects of triplicate fermentations of 24 bean genotypes (four selected from six major market classes) with 12 microbiotas; p-values from the Kruskal-Wallis test were adjusted using the Benjamini-Hochberg method (p.adj).

**Supplementary Table S3. Effect of market class on diversity metrics in terms of Bray-Curtis distance from the fermented blank and Shannon diversity in the population structure study.** Data represent means and standard errors by subject (microbiota) and across all subjects of triplicate fermentations of 24 bean genotypes (four selected from six major market classes) with 12 microbiotas; means within row that are marked with different letters are significantly different (Dunn's test with Holm-Bonferroni-adjusted p<0.05); p-values from the Kruskal-Wallis test were adjusted using the Benjamini-Hochberg method (p.adj).

**Supplementary Table S4. Effect of landrace on microbiota composition at the genus level in the population structure study.** Data represent mean relative abundance and standard error by subject (microbiota) and across all subjects of triplicate fermentations of 24 bean genotypes (four selected from six major market classes) with 12 microbiotas; p-values from the Kruskal-Wallis test were adjusted using the Benjamini-Hochberg method (p.adj); "Other" represents the sum of ASVs that were present in <10% of samples.

**Supplementary Table S5. Effect of market class on microbiota composition at the genus level in the population structure study.** Data represent mean relative abundance and standard error by subject (microbiota) and across all subjects of triplicate fermentations of 24 bean genotypes (four selected from six major market classes) with 12 microbiotas; means within row that are marked with different letters are significantly different (Dunn's test with Holm-Bonferroni-adjusted p<0.05); p-values from the Kruskal-Wallis test were adjusted using the Benjamini-Hochberg method (p.adj); "Other" represents the sum of ASVs that were present in <10% of samples.

**Supplementary Table S6. Effect of landrace and market class on global microbiota composition in terms of Bray-Curtis distance across the Middle American Diversity Panel.** Data represent triplicate fermentations of 299 bean genotypes with three microbiotas; permutational multivariate analysis of variance using distance matrices (PERMANOVA) for landrace and market class by microbiota (subject) and across all subjects.

**Supplementary Table S7. Effect of landrace on diversity metrics in terms of Bray-Curtis distance from the fermented blank and Shannon diversity across the Middle American Diversity Panel.** Data represent means and standard errors by subject (microbiota) and across all subjects of triplicate fermentations of 299 bean genotypes by subject and across all subjects; p-values from the Kruskal-Wallis test were adjusted using the Benjamini-Hochberg method (p.adj).

**Supplementary Table S8. Effect of market class on diversity metrics in terms of Bray-Curtis distance from the fermented blank and Shannon diversity across the Middle American Diversity Panel.** Data represent means and standard errors by subject (microbiota) and across all subjects of triplicate fermentations of 299 bean genotypes with three microbiotas; means within row that are marked with different letters are significantly different (Dunn's test with Holm-Bonferroni-adjusted p<0.05); p-values from the Kruskal-Wallis test were adjusted using the Benjamini-Hochberg method (p.adj).

**Supplementary Table S9. Effect of landrace on microbiota composition at the genus level across the Middle American Diversity Panel.** Data represent mean relative abundance and standard error by subject (microbiota) and across all subjects of triplicate fermentations of 299 bean genotypes with three microbiotas; p-values from the Kruskal-Wallis test were adjusted using the Benjamini-Hochberg method (p.adj); "Other" represents the sum of ASVs that were present in <10% of samples.

**Supplementary Table S10. Effect of market class on microbiota composition at the genus level across the Middle American Diversity Panel.** Data represent mean relative abundance and standard error by subject (microbiota) and across all subjects of triplicate fermentations of 299 bean genotypes with three microbiotas; means within row that are marked with different letters are significantly different (Dunn's test with Holm-Bonferroni-adjusted p<0.05); p-values from the Kruskal-Wallis test were adjusted using the Benjamini-Hochberg method (p.adj); "Other" represents the sum of ASVs that were present in <10% of samples.

**Supplementary Table S11. Effect of landrace on short chain fatty acid production across the Middle American Diversity Panel.** Data represent mean mmol/g bean before digestion and standard error by subject (microbiota) and across all subjects of triplicate fermentations of 299 bean genotypes with three microbiotas; p-values from the Kruskal-Wallis test were adjusted using the Benjamini-Hochberg method (p.adj).

**Supplementary Table S12. Effect of market class on short chain fatty acid production across the Middle American Diversity Panel.** Data represent mean mmol/g bean before digestion and standard error by subject (microbiota) and across all subjects of triplicate fermentations of 299 bean genotypes with three microbiotas; means within row that are marked with different letters are significantly different (Dunn's test with Holm-Bonferroni-adjusted p<0.05); p-values from the Kruskal-Wallis test were adjusted using the Benjamini-Hochberg method (p.adj).

**Supplementary Table S13. Polymicrobial trait loadings.** Genera (loadings) with the most contribution in the first four principal components (PC) and canonical discriminants (CD) for each polymicrobial trait analysis method after *in vitro* fermentation with the MDP common bean genotypes. PC (CD) loadings < -0.4 (< -2.5) are shaded blue and > 0.4 (> 2.5) are shaded red. N/A indicates that all genera had loadings smaller than the cutoffs.

**Supplementary Table S14.** **Variance component decomposition of microbiome features.** Variance components determined by GWAS estimated across all the microbiome features from the three donor microbiomes. Only microbiome features > 0.1 are shown.

**Supplementary Table S15. Summary of microbiome features from the AiMS platform by microbiome.** A broad-sense heritability cutoff of >0.1 was used to determine which mirobiome traits would be used in the genetic association analysis. ASV = amplicon sequence variant; SCFA = short-chain fatty acid.

**Supplementary Table S16. Genome-wide association results summary.** Broad sense heritability (H2) values > 0.10 and highest LOD score per chromosome for each trait from the three donor microbiomes. Bolded values indicate Bonferroni cutoff *p* < 0.05 (LOD > 6.5). Darker color red/green indicates greater LOD score/H2.

**Supplementary Table S17. Descriptions of Multiple Effect Loci.** The single nucleotide polymorphisms (SNPs) and traits from the three microbiomes that had significant associations in the genome-wide association study (GWAS) in each of the seven MEL (-log10(p-value) > 6.5; (Bonferroni correction ⍺ = 0.05)).

**Supplementary Table S18. Population membership and metadata of MDP common bean genotypes used in this study.**

**Supplementary Table S19. MDP common bean genotypes (n = 176) used in the validation study.**
