# Supplementary material for: Genetic variation and historical breeding patterns in common bean (Phaseolus vulgaris L.) affect fermentation patterns by the human gut microbiome
Source: Commun Biol. 2025 Nov 26;8:1690. doi: 10.1038/s42003-025-09089-2 (PMC12657933; doi:10.1038/s42003-025-09089-2)
Supplement: Supplementary file 3 — Reporting Summary [file 42003_2025_9089_MOESM3_ESM.pdf]

nature portfolio

Corresponding author(s): Devin Rose
Andrew Benson

Last updated by author(s): Oct 8, 2025

Reporting Summary

Nature Portfolio wishes to improve the reproducibility of the work that we publish. This form provides structure for consistency and transparency in reporting. For further information on Nature Portfolio policies, see our [Editorial Policies] and the [Editorial Policy Checklist].

Statistics

For all statistical analyses, confirm that the following items are present in the figure legend, table legend, main text, or Methods section.

n/a	Confirmed	
☐	☒	The exact sample size (*n*) for each experimental group/condition, given as a discrete number and unit of measurement
☐	☒	A statement on whether measurements were taken from distinct samples or whether the same sample was measured repeatedly
☐	☒	The statistical test(s) used AND whether they are one- or two-sided<br>*Only common tests should be described solely by name; describe more complex techniques in the Methods section.*
☐	☒	A description of all covariates tested
☒	☐	A description of any assumptions or corrections, such as tests of normality and adjustment for multiple comparisons
☐	☒	A full description of the statistical parameters including central tendency (e.g. means) or other basic estimates (e.g. regression coefficient) AND variation (e.g. standard deviation) or associated estimates of uncertainty (e.g. confidence intervals)
☐	☒	For null hypothesis testing, the test statistic (e.g. *F*, *t*, *r*) with confidence intervals, effect sizes, degrees of freedom and *P* value noted<br>*Give P values as exact values whenever suitable.*
☒	☐	For Bayesian analysis, information on the choice of priors and Markov chain Monte Carlo settings
☐	☒	For hierarchical and complex designs, identification of the appropriate level for tests and full reporting of outcomes
☐	☒	Estimates of effect sizes (e.g. Cohen's *d*, Pearson's *r*), indicating how they were calculated

Our web collection on [statistics for biologists] contains articles on many of the points above.

Software and code

Policy information about [availability of computer code]

Data collection	No special software was used to collect data. Data were recorded in Excel.
Data analysis	R Statistical Software v4.3.0

For manuscripts utilizing custom algorithms or software that are central to the research but not yet described in published literature, software must be made available to editors and reviewers. We strongly encourage code deposition in a community repository (e.g. GitHub). See the Nature Portfolio [guidelines for submitting code & software] for further information.

Data

Policy information about [availability of data]

All manuscripts must include a [data availability statement]. This statement should provide the following information, where applicable:

- Accession codes, unique identifiers, or web links for publicly available datasets
- A description of any restrictions on data availability
- For clinical datasets or third party data, please ensure that the statement adheres to our [policy]

Raw sequence reads from 16S rRNA gene sequencing are available in the Sequence Read Archive under accession number PRJNA1062343.

Research involving human participants, their data, or biological material

Policy information about studies with human participants or human data. See also policy information about sex, gender (identity/presentation), and sexual orientation and race, ethnicity and racism.

Reporting on sex and gender	Sex and gender of fecal donors were reported but not used in the data analysis.
Reporting on race, ethnicity, or other socially relevant groupings	Race or ethnicity of fecal donors were not reported or used in the data analysis.
Population characteristics	Population characteristics of fecal donors were not reported or used in the data analysis.
Recruitment	Participants were recruited through paper advertisements and email communications.
Ethics oversight	20160816311EP

Note that full information on the approval of the study protocol must also be provided in the manuscript.

Field-specific reporting

Please select the one below that is the best fit for your research. If you are not sure, read the appropriate sections before making your selection.

☒ Life sciences ☐ Behavioural & social sciences ☐ Ecological, evolutionary & environmental sciences

For a reference copy of the document with all sections, see nature.com/documents/nr-reporting-summary-flat.pdf

Life sciences study design

All studies must disclose on these points even when the disclosure is negative.

Sample size	For the GWA study, 299 common bean genotypes were analyzed across 3 human gut microbiomes. In the population structure study, 24 bean genotypes were selected across 12 human gut microbiomes. All samples were analyzed in triplicate.
Data exclusions	No data were excluded
Replication	All experiments were replicated either across microbiomes or across genotypes. Technical replications were also run, which were separately run experiments, but bean flours were from the same bag.
Randomization	Common bean genotypes were randomized in an augmented partially replicated design across plates.
Blinding	Samples were coded during analysis. No blinding was implemented during data analysis.

Reporting for specific materials, systems and methods

We require information from authors about some types of materials, experimental systems and methods used in many studies. Here, indicate whether each material, system or method listed is relevant to your study. If you are not sure if a list item applies to your research, read the appropriate section before selecting a response.

Materials & experimental systems

n/a	Involved in the study	
☒	☐	Antibodies
☒	☐	Eukaryotic cell lines
☒	☐	Palaeontology and archaeology
☒	☐	Animals and other organisms
☒	☐	Clinical data
☒	☐	Dual use research of concern
☐	☒	Plants

Methods

n/a	Involved in the study	
☒	☐	ChIP-seq
☒	☐	Flow cytometry
☒	☐	MRI-based neuroimaging

Dual use research of concern

Policy information about dual use research of concern

Hazards

Could the accidental, deliberate or reckless misuse of agents or technologies generated in the work, or the application of information presented in the manuscript, pose a threat to:

No	Yes	
☒	☐	Public health
☒	☐	National security
☒	☐	Crops and/or livestock
☒	☐	Ecosystems
☒	☐	Any other significant area

Experiments of concern

Does the work involve any of these experiments of concern:

No	Yes	
☒	☐	Demonstrate how to render a vaccine ineffective
☒	☐	Confer resistance to therapeutically useful antibiotics or antiviral agents
☒	☐	Enhance the virulence of a pathogen or render a nonpathogen virulent
☒	☐	Increase transmissibility of a pathogen
☒	☐	Alter the host range of a pathogen
☒	☐	Enable evasion of diagnostic/detection modalities
☒	☐	Enable the weaponization of a biological agent or toxin
☒	☐	Any other potentially harmful combination of experiments and agents

Plants

Seed stocks	A total of 299 common bean genotypes representing the Middle American gene pool were used in this study.<br>The Middle American Diversity Panel is described in Moghaddam et al. Plant Genome. 2016;9.
Novel plant genotypes	n/a
Authentication	n/a
